# Supplementary material for: Development of a Health Research Portfolio Based on Priority Topics for Peruvian Social Health Insurance (ESSALUD) in 2023–2025: A Collaborative Approach to Addressing Institutional and Public Health Challenges
Source: Healthcare (Basel). 2025 Feb 27;13(5):514. doi: 10.3390/healthcare13050514 (PMC11898407; doi:10.3390/healthcare13050514)
Supplement: Supplementary file 1 [file healthcare-13-00514-s001.zip › healthcare-3406382-supplementary.pdf]

SUPPLEMENTARY MATERIAL

Table S1. List of Prioritized Topics and Subtopics

TOPIC	SUBTOPIC
**Cancer**	Opportunity for cancer patient care.
	Access to early diagnosis and detection.
	Survival in pediatric and adult cancer patients.
	Epidemiology of cervical cancer.
**Mental Health**	Teleconsultations and teleorientation in mental health.
	Characterization of psychiatric emergency care.
	Depression, anxiety, and pathological grief post-pandemic.
	Common mental illnesses and care at primary and secondary levels.
**Cardiovascular Disease**	Prevention of cardiovascular diseases.
	Operational research and evaluation of interventions.
	Myocardial infarction care networks.
	Characteristics of patients by special groups.
**Diabetes Mellitus**	Metabolic control in the presentation of complications.
	Impact of comprehensive management on prevention and late complications.
	Comorbidities in diabetic foot patients.
	Educational interventions.
**Malnutrition and Anemia**	Effectiveness, safety, and cost-effectiveness of iron supplements in children and pregnant women.
	Eating habits in young children and pregnant women.
	Adherence to iron treatment across age groups.
	Impact of childhood malnutrition (underweight, obesity, overweight).
**Maternal, Perinatal, and Neonatal Health**	Reduction of maternal death.
	Adequate prenatal care.
	Management of preterm birth and premature newborns.
	Breastfeeding promotion.
**Antimicrobial Resistance**	Situational diagnosis of microbiology labs.
	Timely intervention for antimicrobial resistance.
	Implementation of platforms for pathogen identification.
	Rational use of antibiotics and adverse reaction protocols.
**COVID-19, Tuberculosis, and Other Infectious Diseases**	Epidemiology and effectiveness of interventions in nosocomial infections.
	Epidemiology of MDR/XDR tuberculosis.
	Tuberculosis/HIV coinfection.
	Post-COVID conditions and associated disease burden.
**Resource Generation and Financing**	Economic impact of drug and device use.
	Identifying major financial expenditures.
	New funding sources through international cooperation.
	Interoperability of IT systems.
**Service Provision**	Interculturality in health service delivery.
	Quality management focusing on patient safety.
	Impact of home-based and palliative care.
	Implementation of Primary Health Care.
**Management**	Improvement of outpatient and surgical processes.
	Use of unified lab information systems.
	Optimization of technology acquisition.
	Current state of logistics processes and economic impact of delays.
**Digital Health**	Application of IoT for interconnectivity of biomedical equipment.

	Initiatives for hospital service centralization via cloud technology.
	Impact of barriers in patient care system infrastructure.
	Acceptability of telehealth by patients and professionals.

Source: Resolution N°24-IETSI-ESSALUD-2023

Table S2. Evaluation Instrument for Prioritizing Research to be Developed

**Dimension**	**Indicators**	**Scales (Score)**
Time	Is the benefit of this research tangible or observable in the short, medium, or long term?	1: Long term 2: Medium term 3: Short term
Population Coverage	Does this proposal aim to serve a large insured population?	0: 0%-19.9% 1: 20%-39.9% 2: 40%-59.9% 3: ≥60%
Cost-Benefit	Could the application of research results reduce costs for the institution?	0: No 1: Yes
Institutional or External Support	Does the research have international collaborations, strategic partners, or external researchers considered for the proposal? (Intra-institutional, National agreements URP, UTEC, etc., International agreements)	1: International 2: National university or institution 3: Intra-institutional
Resource Feasibility	Is the proposed research realistic in terms of resources? (Access to data, Qualified personnel, Equipment, Financing) (One point per component, cumulative)	-
Data Feasibility	Is there immediate availability of data?	0: No 1: Yes
Scope of Results	Does it have national, Lima Metropolitan and Callao, or Independent Networks coverage?	1: Local 2: Regional 3: National
Innovation	Does the proposed research contribute something new to the field, have the potential to change clinical practice or health policy, or challenge current methods?	0: No 1: Yes
Results	What results are expected from the research? (One point per component, cumulative)	01 report for decision-making, 01 device or hardware, 01 publication, 01 technological package, 01 method for diagnosis, screening, prognosis, monitoring, or treatment, 01 process innovation, 01 software program

Source: Prepared by the Health Research Directorate of IETSI-ESSALUD
